# The complete chloroplast genome of *Impatiens mengtszeana* (Balsaminaceae), an endemic species in China

**DOI:** 10.1080/23802359.2021.1994894

**Published:** 2022-02-15

**Authors:** Baizhu Li, Yu Li, Zhaofeng Li, Mengda Xiang, Xi Dong, Xiaoxin Tang

**Affiliations:** a Key Laboratory of National Forestry and Grassland Administration on Biodiversity Conservation in Karst Mountainous Areas of Southwestern China, School of Life Science, Guizhou Normal University, Guiyang, China; b Key Laboratory of Plant Physiology and Developmental Regulation, School of Life Science, Guizhou Normal University, Guiyang, China

**Keywords:** *Impatiens mengtszeana*, endemic species, chloroplast genome, phylogenetic analysis

## Abstract

*Impatiens mengtszeana* is an endemic species in China. In this study, the complete chloroplast genome of *I. mengtszeana* was sequenced and analyzed. The total chloroplast genome size of *I. mengtszeana* is 152,928 bp, including a pair of inverted repeat regions (IRs, 26,007 bp) separated by a large single copy (LSC, 83,722 bp) region and a small single copy region (SSC, 17,192 bp). The whole chloroplast genome contains 89 protein-coding genes (PCGs), 37 transfer RNA genes (tRNAs), and eight ribosomal RNA genes (rRNAs). According to the phylogenetic topologies, *I. mengtszeana* was closely related to *I. hawkeri*.

*Impatiens* is a large genus in the family Balsaminaceae, comprises over 1000 species, mainly distributed in tropical and subtropical regions (Yu et al. 2016; Stefaniak et al. 2020). It is known for its multi-colors flowers and ornamental values. The chemical substances in the rhizome of *Impatiens* are widely used in the field of medicine (Zhou et al. 2015; Chua 2016). *Impatiens* is considered to be one of the most taxonomically difficult groups in angiosperm. The species within the genus are extremely variable and lack the synapomorphic characters necessary to define subdivisions. (Grey-Wilson 1980; Yu 2012). *Impatiens mengtszeana* is an annual herb, endemic to south-west China (Lan et al. 2004; Yu 2012). Flowers are light yellow with red streak. At present, there are few studies on *I. mengtszeana*. Herein, it is necessary to describe evolutionary relationships in terms of relative species of *Impatiens* based on highthroughput sequencing approaches. This study provides important information of Balsaminaceae on evolution, genetic and molecular biology for future studies.

The fresh leaves of *I. mengtszeana* were sampled from Malipo County, Wenshan City, Yunnan Province, China (104°50′39.73″ E, 23°10′9.07″ N, 1420 m altitude) and DNA samples were stored in Guizhou Normal University (accession number: TR-010). Total DNA was extracted from fresh leaves by CTAB method and applied to 500-bp paired-end library construction using the TruSeq Nano DNA Sample Prep Kit for Illumina sequencing. Sequencing was carried out on the Illumina NovaSeq 6000 platform (BIOZERON Co., Ltd, Shanghai, China). Approximately 2.4 Gb of raw data from *I. mengtszeana* were generated with 150 bp paired-end read lengths. The low quality sequences of raw reads used Trimmomatic 0.39 for quality control (Bolger et al. 2014). The trimmed reads were selected for cp genome assembly by NOVOPlasty4.2 software (Dierckxsens et al. 2017). The complete chloroplast genome of *I. mengtszeana* was annotated by GeSeq (Tillich et al. 2017). For a better understanding of *I. mengtszeana*, the physical map of the new chloroplast genome was generated using OGDRAW (Lohse et al. 2013). The complete cp genome was submitted to Genbank (accession number of MW727522).

The complete cp genome sequence of *I. mengtszeana* is 152,928 bp and shows a characteristic circular structure. Overall GC content of the whole genome is 36.73%. A large single-copy (LSC, GC-34.34%) region of 83,722 bp and a small single-copy (SSC, GC-29.53%) region of 17,192 bp, which is separated by a pair of inverted repeat (IRa and IRb) regions of 26,007 bp. There are a total of 89 protein-coding genes, 37 tRNA genes, and eight rRNA genes. Among these, duplication events occurred with 19 genes (rps12, rp12, rp123, ycf2, ycf15, ndhB, rps7, ycf1, trnA-UGC, trnG-GCC, trnI-CAU, trnI-GAU, trnL-CAA, trnN-GUU, trnV-GAC, rrn16, rrn23, rrn4.5, rrn5).

In order to further determine the phylogenetic location of *I. mengtszeana*, 20 cp genome sequences from Ericales were downloaded from GenBank. BioAider1.0 software was used for the alignment of cp genome sequences (Zhou et al. 2020). A neighbor-joining phylogenetic tree was constructed in MEGA7 software with 1000 bootstrap replicates (Kumar et al. 2016). The NJ tree analysis indicated that *I. mengtszeana* is closely related to *Impatiens hawkeri* with 100% bootstrap support (Figure 1).

**Figure 1.**
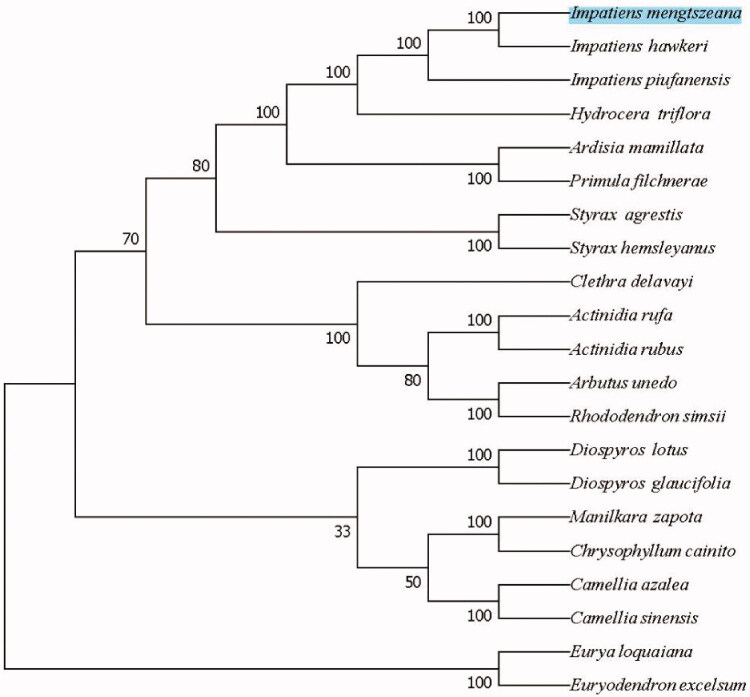
The NJ phylogenetic tree for *I. mengtszeana* based on 20 chloroplast genome sequences of Ericales. Numbers on the nodes are bootstrap values from 1000 replicates. Accession numbers: *Actinidia rufa* (NC_039973.1), *Actinidia rubus* (MN652056.1), *Clethra delavayi* (NC_041129.1), *Impatiens hawkeri* (NC_048520.1), *Impatiens piufanensis* (NC_037401.1), *Hydrocera triflora* (NC_037400.1), *Diospyros lotus* (NC_030786.1), *Diospyros glaucifolia* (NC_030784.1), *Arbutus unedo* (JQ067650.2), *Rhododendron simsii* (MW030509.1), *Eurya loquaiana* (NC_050937.1), *Euryodendron excelsum* (NC_039178.1), *Primula filchnerae* (NC_051972.1), *Ardisia mamillata* (MN136062.1), *Manilkara zapota* (MN295595.1), *Styrax agrestis* (MT644192.1) and *Styrax hemsleyanus* (NC_047298.1).

## Data Availability

The data that support the findings of this study are openly available in NCBI at https://www.ncbi.nlm.nih.gov/nuccore/ MW727522, reference number MW727522.
